# Identification of a Novel Pathogenic Rearrangement Variant of the APC Gene Associated with a Variable Spectrum of Familial Cancer

**DOI:** 10.3390/diagnostics11030411

**Published:** 2021-02-28

**Authors:** María Lourdes Garza-Rodríguez, Víctor Treviño, Antonio Alí Pérez-Maya, Hazyadee Frecia Rodríguez-Gutiérrez, Moisés González-Escamilla, Miguel Ángel Elizondo-Riojas, Genaro A. Ramírez-Correa, Oscar Vidal-Gutiérrez, Carlos Horacio Burciaga-Flores, Diana Cristina Pérez-Ibave

**Affiliations:** 1 Centro Universitario Contra el Cáncer (CUCC), Servicio de Oncología, Universidad Autónoma de Nuevo León, Hospital Universitario “Dr. José Eleuterio González”, Nuevo, León 64460, Mexico; lulugarza87@gmail.com (M.L.G.-R.); hazyadee@gmail.com (H.F.R.-G.); drmoisesgzz87@gmail.com (M.G.-E.); riojas_miguel@hotmail.com (M.Á.E.-R.); vidal_oscar@hotmail.com (O.V.-G.); 2 Tecnológico de Monterrey, Escuela de Medicina y Ciencias de la Salud, Nuevo, León 64710, Mexico; vtrevino@itesm.mx; 3 Departamento de Bioquímica y Medicina Molecular, Facultad de Medicina, Universidad Autónoma de Nuevo León, Nuevo, León 64460, Mexico; bioquimicomty@gmail.com; 4 Department of Molecular Science, UT Health Rio Grande Valley, McAllen, TX 78502, USA; genaro.ramirezcorrea@utrgv.edu; 5 Department of Pediatrics, Division of Cardiology, Johns Hopkins University School of Medicine, Baltimore, MD 21205, USA

**Keywords:** familial adenomatous polyposis (FAP), adenomatous polyposis coli (*APC*), pathogenic germline variant, rearrangement

## Abstract

Familial adenomatous polyposis (FAP) is an autosomal-dominant condition characterized by the presence of multiple colorectal adenomas, caused by germline variants in the adenomatous polyposis coli (*APC*) gene. More than 300 germline variants have been characterized. The detection of novel variants is important to understand the mechanisms of pathophysiology. We identified a novel pathogenic germline variant using next-generation sequencing (NGS) in a proband patient. The variant is a complex rearrangement (c.422+1123_532-577 del ins 423-1933_423-1687 inv) that generates a complete deletion of exon 5 of the *APC* gene. To study the variant in other family members, we designed an endpoint PCR method followed by Sanger sequencing. The variant was identified in the proband patient’s mother, one daughter, her brother, two cousins, a niece, and a second nephew. In patients where the variant was identified, we found atypical clinical symptoms, including mandibular, ovarian, breast, pancreatic, and gastric cancer. Genetic counseling and cancer prevention strategies were provided for the family. According to the American College of Medical Genetics (ACMG) guidelines, this novel variant is considered a PVS1 variant (very strong evidence of pathogenicity), and it can be useful in association with clinical data for early surveillance and suitable treatment.

## 1. Introduction

Colorectal cancer (CRC) is a significant cause of morbidity and mortality globally [1]. CRC is the third most commonly diagnosed cancer and the second in mortality, with 1.8 million new cases and almost 861,000 deaths in 2018, according to the GLOBOCAN database of the World Health Organization (WHO). In Mexico, during 2018, CRC incidence was 149,000 people, with mortality of 70,841 [2].

About 85% of all CRC cases are considered sporadic. Hereditary forms of CRC represent up to 15% of all cases and 10% correspond to “familial” cancers, followed by 5% of hereditary cancer syndromes (HCSs). Hereditary cancer syndromes include nonpolyposis cancer (HNPCC or Lynch syndrome: ~3–5%) and various polyposis syndromes (~1–2%) such as familial adenomatous polyposis (FAP), familial juvenile polyposis (JP), MUTHY-related polyposis, and Peutz–Jeghers syndrome [1]. FAP is an autosomal-dominant condition that affects almost 1 in 5000 people and represents about 1% of all CRC cases [3]. FAP is a precancerous condition that is clinically characterized by the presence of multiple colorectal adenomas or polyps found in the walls of the large intestine and the rectum [4,5,6]. Classical FAP usually develops more than 100 polyps, 10 to 100 in its attenuated form and a profuse form in which there are more than 1000 polyps [7]. The risk of cancer is practically 100% if the polyps are not detected and removed by the time they are 40 years old [1,8]. Prophylactic colectomy, which is the surgical removal or resection of a diseased part of the colon, has been recommended for these patients [4]. Due to the high risk of developing colon cancer, 7% of untreated patients will develop colon cancer by age 21, 87% at 45 years, and 93% by 50 [5].

Symptoms are rare in children and adolescents until the polyps are large and numerous, causing rectal bleeding or even anemia. Other nonspecific symptoms include constipation or diarrhea, abdominal pain, palpable abdominal masses, and weight loss in young patients. Extraintestinal manifestations such as osteomas, dental abnormalities, congenital hypertrophy of the retinal pigment epithelium (CHRPE), desmoid tumors, and extracolonic cancers may also occur (thyroid, liver, bile ducts, and central nervous system) [1].

The FAP diagnosis is made considering several factors: phenotype, family history, medical records, and next-generation sequencing of the adenomatous polyposis coli (*APC*) gene. This inherited syndrome is due to germline variants in the *APC* gene (OMIM 175100) [4], which is a tumor suppressor gene [9] located on the long arm of chromosome 5 in band q21. This gene contains 21 exons within a 98 kb locus [10]. The APC gene spans a region of 138,742 bp (NC_000005) [1], and the coding region contains 15 exons in an 8535 nucleotide region encoding a 2843 amino acid multidomain protein with a molecular weight of 310 kDa [9,11]. The largest exon is exon 15, with a remarkable 6579 bp uninterrupted open reading frame, comprising more than 75% of this gene’s coding sequence [10].

The APC protein plays an essential role in the cell cycle, motility, adhesion, and signaling. It is often referred to as a multitasking protein, and the disruption of its interactions can lead to APC’s inability to perform these functions, thereby contributing to tumor formation. APC’s primary functions are downregulation of the Wnt pathway (via β-catenin), modulation of cell adhesion/migration, and chromosomal stability maintenance [10].

Based on information from the Leiden Open Variation Database, 1801 unique allelic variants of the *APC* gene are included (information retrieved on 01/28/2021) [12], and more than 90% of variants produce a truncated protein as a result of a stop codon, especially at the C-terminal domain [10]. The most common variant occurring in approximately 10% of patients with FAP is a deletion at codon 1309 (exon 15); another common variant is a deletion at codon 1061 (exon 15), occurring in 5% of patients [1]. Deletions in codon 1309 (exon 15) have been identified in patients with no family history, suggesting that they may also arise de novo. Germline variants can predict the phenotype due to its location within the *APC* gene [10,13]. A phenotype of multiple polyps (more than 5000) is predicted when the variant arises between codon 1249 and codon 1330 of exon 15. The profuse FAP phenotype involved codon 232 of exon 7 and codons 805, 1062, 1104, and 1309 of exon 15. Variants in codon 1309 correlate with cancer aggressiveness ([13]), whereas variants upstream or downstream of this region result in a phenotype of fewer than 1000 polyps [12]. Attenuated FAP (AFAP) is caused by variants at codons 78 (exon 3) to 163 (exon 4) at the 5′ end. This disease is characterized by the development of very few polyps (less than 100).

Similarly, germline variants in exon 15 are also associated with an attenuated phenotype [13]. Additionally, the AFAP phenotype has been correlated with variants in three regions of *APC*: at the 5′ end (the first five exons), in the alternatively spliced region of exon 9, or at the 3′ end (after codon 1580) [14]. Germline variants between codons 1395 and 1560 (exon 15) have also been associated with desmoid tumors and mandibular osteomas [10]. There is a correlation between the variants’ site and the clinical phenotype; however, inter- and intrafamilial variability has been reported [15]. An increasing number of new variant patterns have been reported for some monogenic disorders, including FAP [16]. The identification of new variants has led to the characterization of hereditary syndromes; however, there are still rare variants beyond the classical clinical spectrum. Therefore, it is an obligation of all investigators to describe all novel variants to fulfill these clinical gaps.

This study presents a Mexican family’s molecular analysis in which FAP is due to a novel germline variant in the *APC* gene. Once we found the variant in the proband by next-generation sequencing (NGS), we developed a low-cost endpoint PCR method followed by Sanger sequencing to detect the other family members’ germline variant. Herein we describe the family’s clinical history, the design of a molecular detection method, and the identification of carriers.

## 2. Materials and Methods

### 2.1. Approval from the Scientific and Ethics Committees

The study was conducted in the Centro Universitario Contra el Cáncer (CUCC), at the CUCC Early Cancer Detection Clinic (CECIL), in accordance with the Declaration of Helsinki. The Institutional Ethics Committee approved the protocol of the University Hospital (Registration No.: ON18-00015). All patients were invited to participate in the research project, an interview was performed, and once the patients agreed to participate, they signed informed consent. Afterwards, clinical and epidemiological information was collected, and blood samples were withdrawn.

### 2.2. Case Presentation

The proband patient was a 49-year-old healthy woman who attended the CECIL consultation because of a family history of cancer; she is the first child of nonconsanguineous parents. The clinical diagnosis was based on the patient’s family history: a brother with mandibular cancer at 44, a cousin with colon polyps at 44, the mother had ovarian cancer at 62, a maternal aunt deceased by pancreatic cancer at 73, grandfather deceased by colon cancer at 82, great uncle with colon cancer at 80, cousin aunt with breast cancer at 40, and cousin aunt deceased by gastric cancer at 59. Additionally, two nieces have glycogenosis type I.

As a proband patient’s pathological clinical history, she had a hernioplasty at the age of 45, no prescribed medications, menarche at 12, irregular menstruation, was sexually active since age 29, had Gravida 2 times, and had 2 term pregnancies with normal vaginal delivery. Her previous cancer prevention studies were a Papanicolau test (normal) and a mammography (BIRADS 3). She met the clinical criteria for HCS and received genetic counseling. After the genetic consultation, she was diagnosed with probable Lynch syndrome, and the clinical team recommended a colonoscopy. She returned four months later with a colonoscopy finding of less than 10 adenomatous polyps, all removed and analyzed without malignancy. Additionally, she shared new familial information: she informed us that her brother developed an oral cavity epidermoid carcinoma, and her maternal aunt developed multiple adenomatous polyps in the colon (>10 polyps), duodenum, and stomach (<10 polyps) at the age of 64 and underwent total colectomy because of the polyposis; some of the polyps that were analyzed revealing mismatch microsatellite repair (MMR) stability. With the new data, the diagnosis changed to probable familial polyposis syndromes: FAP or MUTYH-related.

### 2.3. Proband Patient NGS Study

Buccal swab samples were collected from the proband patient. Genetic testing with a 30-gene NGS panel (Onco Life Test^®^) from Life in Genomics^®^ (Mexico City, Mexico) was realized. The panel includes 30 genes for breast and ovarian cancer syndrome (HBOC), hereditary nonpolyposis colon cancer (HNPCC), Lynch syndrome, FAP, Cowden syndrome, and Peutz–Jeghers syndrome, as well as for prostate, stomach, pancreas, and fallopian tube cancers. A pathological variant in the *APC* gene (c.422 + 1123_532-577 del ins 423-1933_423-1687 inv) was found. The novel pathogenic variant was not previously reported in the ClinVar database from NCBI. This variant results in a complete deletion of exon 5, keeping the splicing sites intact. Once the variant was identified in the proband patient, a low-cost endpoint PCR–Sanger sequencing method was designed to analyze the samples from 10 relatives.

### 2.4. DNA Extraction and Quantification

Peripheral blood samples (5 mL) from the proband patient and her relatives were collected by venipuncture. Samples were centrifuged at 1850× *g* for 10 min at room temperature. Genomic DNA was isolated from leukocytes using the QIAamp DNA Blood Midi kit (QIAGEN, Hilden, Germany) following the manufacturer’s recommendations.

DNA was quantified by measuring the optical density (OD) at 260 nm using the QIAxpert UV/Vis spectrophotometer (QIAGEN, Hilden, Germany). The ratio OD260/OD280 determined DNA purity; values between 1.8 and 2 were considered pure. The genomic DNA was stored at −80 °C until use.

### 2.5. Primer Design and PCR

**Figure 1:**
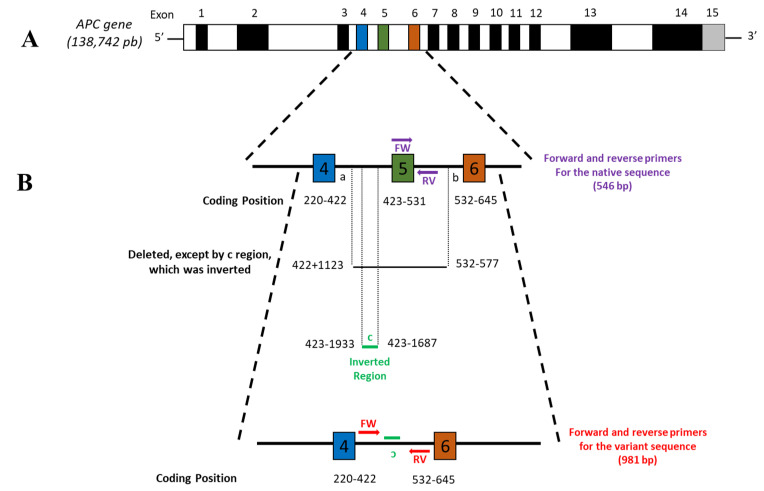
*APC* gene and variant analysis. (**A**) Native *APC* gene structure. The gene is located on chromosome 5q21-22 with a size of 138,742 bp. The coding region contains 15 exons, represented by dark and color regions (not at scale). (**B**) Novel *APC* variant structure (c.422+1123_532-577 del ins 423-1933_423-1687inv). The region from “a” to “b” is shown, including exon 5. The fragment from “a” to “b” is deleted, completely taking exon 5 with it. Nevertheless, within fragment “a-b,” fragment “c” is inserted inverted. The insert “c” is a portion of the intron between exon 4 and 5, upstream from the beginning of exon 5 from nucleotide 1687. Forward (FW) and reverse (RV) primers that amplify the native and variant regions are shown in purple and red arrows, respectively.

Since we were unable to obtain the NGS reference sequence, we constructed the sequence of the mutated region based on the variant reported by Life in Genomics^®^ (c.422 + 1123_532-577 del ins 423-1933_423-1687 inv). Oligo Primer analysis software v7 by Molecular Biology Insights, Inc. (Cascade, CO, USA) [16] was used to design the specific primers for each *APC* sequence (native and variant). We developed a pair of primers to amplify the variant *APC* allele, the intronic regions that flank the inverted/deleted fragment (the variant sequence generated by the rearrangement). The primers are the following: forward 5′-GCGATTCTTCTGCTTCAGTC-3′, reverse 5′-ACACTTATTTGCCAAAGTCAC-3′ (amplification fragment of 981 bp). To amplify the wild-type *APC* allele, we designed a pair of primers that amplify a region of exon 5 and an underlying 3′ intronic region: forward 5′-TGGTATTACGCTCAACTTC-3′ and reverse 5′-CAAAACAAACCAGCTTAG-3′ (amplification fragment of 546 bp) (Figure 1).

### 2.6. Native and Variant Regions PCR

The PCR was performed in a total volume of 50 µL using 100 ng of genomic DNA, 25 µL of GoTaq^®^ Colorless Master mix 2X from Promega (Fitchburg, WI, USA) and 0.2 mM of each primer. The PCR reaction was carried out in a SimpliAmp™ 96-well Thermal Cycler (Thermo Fisher Scientific, Waltham, MA, USA). The amplification programs used consisted of an initial hold at 95 °C for 5 min, followed by 30 cycles, each including incubations at 95 °C for 1 min. The alignment temperature for wild-type *APC* was 51 °C for 1 min, and the *APC* variant was 61.5 °C for 1 min and 72 °C for 1 min. Finally, an elongation step at 72 °C for 7 min was performed. PCR’s amplification products were visualized on 2% agarose gels stained with SYBR™ Safe DNA Gel Stain from Thermo Fisher Scientific (Waltham, MA, USA) under UV light.

### 2.7. Sanger Sequencing

To validate the method, we verified all the amplified products’ sequences by the Sanger sequencing method. The PCR products were purified with Wizard^®^ SV Gel and PCR Clean-Up System from Promega (Fitchburg, WI, USA) following the manufacturer’s instructions. The PCR products were subsequently subjected to sequencing with BigDye terminator v1.1 cycle sequencing reagents (Applied Biosystems, Foster City, CA, USA), purified with BigDye XTerminator™ purification kit (Applied Biosystems, Foster City, CA, USA) according to the manufacturer’s recommendations, and analyzed on an ABI 3130 genetic analyzer (Applied Biosystems, Foster City, CA, USA).

### 2.8. Bioinformatics Analysis

We used Sequencing Analysis v5.2 and SeqScape v2.6 software (Applied Biosystems, Foster City, CA, USA) to verify the sequences.

## 3. Results

In this study, we detected a novel variant of the *APC* gene in a family with FAP from Monterrey, Mexico, that attended our clinic (CECIL). The proband (III-4) was a 49-year-old woman, and based on clinical examination, family history, and genetic counseling, she was diagnosed as probable Lynch syndrome or MUTYH-related. The clinical team suggested a colonoscopy. The results showed the presence of polyps and tied her to two relatives who developed neoplasms: her brother developed an oral cavity epidermoid carcinoma, and her 64-year- old maternal aunt developed multiple adenomatous polyps in the colon, duodenum, and stomach. With these new data, the diagnosis changed to a familial polyposis syndrome, such a FAP or MUTYH-related. An NGS panel was performed (OncoLife Test^®^) on the index patient, and we identified a new pathological variant in the *APC* gene (c.422+1123_532-577 del ins 423-1933_423-1687inv) that generates a complete deletion of exon 5 (Figure 1); this is the reason why the definitive diagnosis for this patient was FAP. The variant was not previously reported in the databases (ClinVar and OMIM). This novel pathogenic variant was submitted in the ClinVar database by the Variation ID 988590 (https://www.ncbi.nlm.nih.gov/clinvar/variation/988590/, accessed on 7 December 2020).

**Figure 2:**
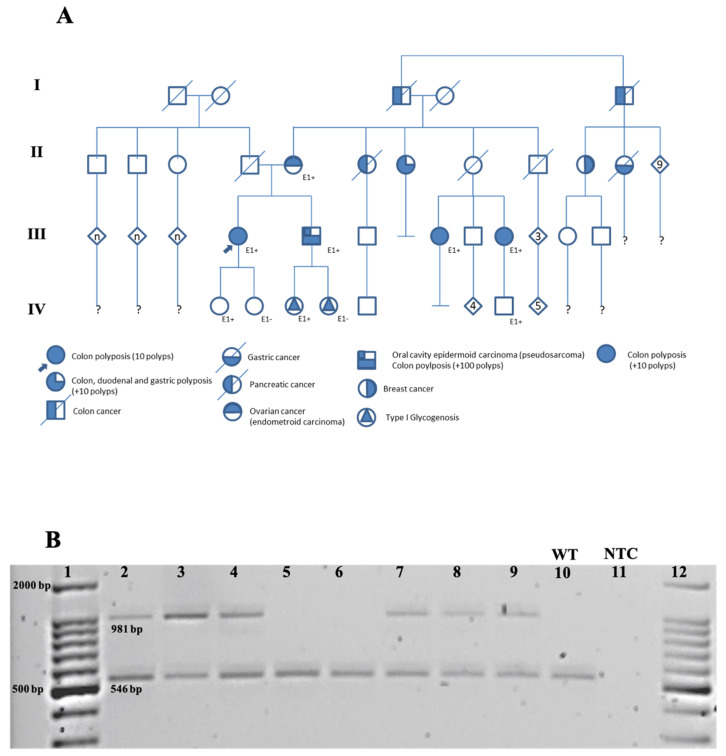
Detection of the *APC* variant in the family. (**A**) Pedigree of the family. Pedigree of the family carrying the novel *APC* variant (c.422+1123_532-577 del ins 423-1933_423-1687inv) is shown. An autosomal dominant pattern of inheritance is observed in four generations. General symbols: the proband is denoted with a filled arrowhead; squares are males; circles are females; diamonds represent a group of relatives of unknown gender; all symbols with a diagonal are deceased; open symbols are clinically unaffected; solid symbols indicate specific pathologies described at the bottom of the pedigree diagram; letter E shows the patients that were tested for the *APC* variant by PCR assay; the plus or minus (+ or −) sign indicates the presence or absence of this variant; the letter “n” is known offspring(s) without a specified number of members, a question mark describes that the offspring is unknown. (**B**) Agarose gel electrophoresis for PCR products. The variant *APC* allele generates an amplified product of 981 bp, and the native *APC* allele amplifies a product of 546 bp. Lanes (1 and 12), molecular weight marker: we found that the index patient (2), her mother (3), one of the daughters (4), a niece (7), a cousin (8), and a son (9), were heterozygous for the pathogenic variant. A daughter (5), a niece (6), and a cousin of the index patient (10) were homozygous for the native *APC* gene. A no-template control (NTC) is shown in Lane 11.

We charted a pedigree of the family (Figure 2A) of four generations with a total of 30 people. The pedigree included two patients with colon cancer, the proband’s maternal grandfather (I-3) and great uncle (I-5); one patient with ovarian cancer, the proband’s mother (II-5); one patient with pancreatic cancer, the proband’s aunt (II-6); one patient with colon, duodenal, and gastric polyposis (>10 polyps), the proband’s uncle (II-7); one patient with breast cancer, the proband’s aunt (II-10); one patient with gastric cancer, the proband’s aunt (II-11); three patients with colon polyposis, the proband patient (III-4) and two maternal cousins (III-7 and 9); one patient with oral cavity epidermoid carcinoma and colon polyposis (III-5), the proband’s brother; and two patients with type I glycogenosis, the proband’s nieces (IV-6 and 7).

Due to the proband patient’s interest in confirming the diagnosis in other family members and their socioeconomic status, using NGS was not an option. Therefore, we designed a method to detect the variant based on endpoint PCR and Sanger sequencing. We assembled the mutated allele sequence manually based on the APC gene sequence reported by the NCBI database (NG_008481.4 RefSeqGene). To create the variant allele, we relied on the *APC* gene sequence published in the GenBank database (Gene ID: 324, Ensembl: ENSG00000134982 MIM: 611731). From the 3′ end of exon 4 of the APC gene (nucleotide 422), we counted upstream 1123 nucleotides, being the a-end of the “a-b” fragment shown in Figure 1. Later at the 5′ end of exon 6 of the *APC* gene (nucleotide 532), 577 nucleotides were counted downstream; this was the b-end of the fragment. The “a-b” fragment was deleted entirely. For fragment “c,” from the 5′ end of exon 5 of the *APC* gene (nucleotide 423), we counted 1933 nucleotides upstream with this position being the 5′ end of fragment “c.” We then counted 1687 nucleotides upstream of the 5′ end of exon 5 of the *APC* gene (nucleotide 423); this is the 3′ end of fragment “c.” We inverted this fragment and pasted it to the *APC* gene sequence to create the variant allele reference sequence (Figure 1).

We performed an endpoint PCR analysis using the primer sets designed for each one (native and variant) to amplify the *APC* alleles. First, we standardized the PCR reaction using the sample from the index patient as a positive control. As a negative control, we used the sample from a healthy individual not related to the family (data not shown). Some of the ten members of the family analyzed were included in Figure 2B. We observed a single band of 546 bp for wild-type homozygous patients, and for the mutated heterozygous patients, we predicted and observed two bands, a 981 bp band and a 546 bp band (Figure 2B). First- and second-degree relatives were analyzed; thus, ten family members were analyzed for the *APC* variant by PCR assay (indicated by the letter E in Figure 2A; the plus or minus sign indicates the presence or absence of this variant).

**Figure 3:**
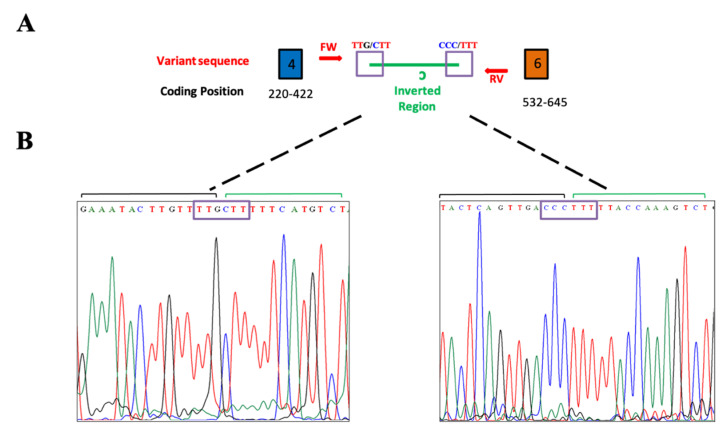
Validation of the sequence for the *APC* variant. (**A**) Amplified fragment. The blue box represents exon 4, the orange box represents exon 6, the red arrows represent the FW and RV primers, and the inverted fragment is in green. The purple squares enclose the region of the insert junction with the intronic region. (**B**) Electropherograms. The two electropherograms show the segments in the junction at the 5′ and 3′ ends of the inverted fragment (ins 423-1933_423-1687inv) with the intronic regions.

We confirmed the variant in the proband (III-4) and the family members by the PCR/Sanger method (Figure 3). We also found the variant in the other seven members: her mother, a daughter, a brother, two cousins, a niece, and a second-degree nephew (II-5, III-5, III-7, III-9, IV-4, IV-6, and IV-11). Two relatives were negative for the *APC* variant, one of her daughters and one niece (IV-5 and IV-7) (Figure 2), both without clinical data of FAP.

The proband patient’s genetic counseling suggested total colectomy as a cancer prevention strategy. A colonoscopy was performed on her brother, who was a carrier of the variant. More than 100 polyps were found in the colon, and he decided to undergo a total colectomy. The niece that carries the variant in *APC* also has confirmed glycogenosis (homozygous pathogenic variant G6PC c.379_380TA/ c.379_380TA), so long-term follow-up is under a multidisciplinary team.

## 4. Discussion

Based on information from the Leiden Open Variation Database (LOVD) database, 1801 unique allelic variants of the *APC* gene are included in the database (information retrieved on 01/28/2021) [12]. These variants are heterogeneous and are associated with colon polyposis in the case of being pathogenic or likely pathogenic. Still, there are also variants of uncertain significance (VUS) and likely benign ones that do not affect polyp development. According to this classification, among the pathogenic variants, most are frameshift variants and nonsense variants, with a few larger deletions or splice variants [15,17,18]. In a review of 431 cases of the Mayo Clinic, 85% of cases were either frameshift or nonsense, and only 6% were deletions [17]. In one Brazilian cohort, most of the patients with more than 1000 polyps had *APC* gene deletion in 42% and nonsense mutations in 28.5%. Additionally, in patients with less than 100 polyps, 50% was due to missense mutations [14]. The majority of these variants lead to premature truncation of the APC protein. Most of the germline variants cause loss of the b-catenin level regulating domain, axin-binding domain, C-terminus microtubule, and EB1-binding domains [19]. Regarding the position, 60% of all variants in *APC* fall in the 5′ portion of the gene [18,20]. According to the *APC* variant database, the 5 bp deletion (c.3927-c.3931 delAAAGA), known as the codon 1309 (exon 15) termination variant, is the most common germline variant detected [21]. The 1309 variant is associated with the classic form of FAP with more than 100 polyps in the colon and early colorectal cancer. Variants between codons 1250 to 1464 (exon 15) are associated with profuse polyposis, the most severe form of FAP. Variants spanning exons 1–5, exon 9, the 3′ region of exon 15, and large interstitial 5q deletions are associated with an attenuated form of FAP (from 10 to 100 polyps) [21].

FAP signs include colonic polyps and extracolonic manifestations, and these are more frequent between codons 976 to 1067, followed by 1310 to 2011 (this region comprises exon 15). Variants between codons 543 (exon 12) and 1309 (exon 15) are associated with congenital hypertrophy of retinal pigment epithelium (CHRPE), whereas variants between codons 1310 to 2011 have a six-fold risk of developing desmoid tumors compared to codons 159 (exon 4) to 495 (exon 11). Duodenal adenomas, which are the second most frequent in which polyps develop in FAP, are usually associated with variants between codons 279 (exon 7) and 1309 (exon 15) but can have a three to four times greater chance of appearing if the variants are between codons 976 and 1067 (exon 15) [22,23]. Another series reported that the most common extracolonic manifestations were upper gastrointestinal polyps (79%) and desmoid tumors (57%); in the latter, the variants were located between codons 1444 to 2843 [14]. In a cohort of South Asian families, 45% presented extracolonic manifestations, the most common was CHRPE in 23.3% and desmoid tumors in 21.6%, with variants in codons 1483 and 1228, respectively [24].

As seen before, depending on the gene’s site and the type of variant, severity can vary; however, it also depends on inter- and intrafamilial variability. These show us that the molecular mechanism that unveils the genotype–phenotype correlation is yet to be fully understood [15,18].

It is interesting to analyze the variant found in this family (c.422 + 1123_532-577 del ins 423-1933_423-1687 inv) because it was not previously reported, and it does not fall in the most frequent type of variant according to the Mayo Clinic Registry. The variant is a complex rearrangement of various gene sections involving a deletion typically associated with attenuated phenotype and one insertion and one inversion. Based on the complex rearrangement of this variant and according to the criteria for classifying pathogenic variants of the ACMG standards and guidelines, the evidence of pathogenicity is robust (PVS1) [25]. PVS1 variants include null variant (nonsense, frameshift, canonical +/−1 or two splice sites, initiation codon, single or multiexon deletion) in a gene where the loss of function is a known mechanism of disease [25,26].

Knowing the molecular mechanisms of the genotype–phenotype is still a challenge; however, despite the high variability in symptoms observed in this family, clinical data can help us classify this as a novel pathogenic variant (PVS1 variant). The age of onset of polyps and carcinomas is generally around the fifth decade of life. Regarding the numbers of polyps, we saw high variability with some of the patients with scarce polyps. This evidence supports that the variant could be associated with an attenuated form of FAP. However, as previously mentioned, there is conflicting evidence in the genotype–phenotype and variable phenotype expression in this disease. There are contradictions in the literature against the sole use of genotype in making clinical decisions. The studies of FAP families are still limited and are noticeable for their contradictory findings. Multiple studies note intra- and interfamily variation in the FAP phenotype in patients carrying the same mutation [19]. Therefore, functional analysis tests are required to determine this germ variant’s mechanism in the *APC* gene. Another interesting finding is the different neoplasms in the family members that carry the variant. There are several reports of double heterozygous families and individuals caring variants in genes related to HCS such as *APC*/*MSH2* in juvenile-onset of colorectal cancer [27], BRCA1/2 in the Ashkenazy Jewish population with breast cancer [28], *APC*/*MLH1* in multiple jejunum cancer [29], and *APC*/*BRCA1* in an Italian family with profuse FAP. This last one is very similar to our case, so the NGS panel test in the mother of the proband (with ovarian cancer) could lead us to a better understanding of this case [30]. Because of this, the management of the family should include screening for other types of cancer. Since there is an atypical tumor spectrum, we speculate the presence of another pathogenic variant in some modifier gene of low penetrance or another unspecified locus.

To date, there are few reports of germline variability of the *APC* gene in the Mexican population. Understanding human genomic variation is a central focus of medical and population genomics.

## Data Availability

This novel pathogenic variant was submitted in the ClinVar database by the following variation ID 988590 (https://www.ncbi.nlm.nih.gov/clinvar/variation/988590/, accessed on 7 December 2020).
